# A group randomized control trial to test the efficacy of the Road to Mental Readiness (R2MR) program among Canadian military recruits

**DOI:** 10.1186/s12888-019-2287-0

**Published:** 2019-10-29

**Authors:** Deniz Fikretoglu, Aihua Liu, Anthony Nazarov, Kristen Blackler

**Affiliations:** 1 Defence Research and Development Canada – Toronto Research Centre, 1133 Sheppard Ave West, Toronto, Ontario M3K 2C9 Canada; 2 Department of Psychiatry, McGill University, Montreal, Quebec, Canada; 3 The MacDonald Franklin OSI Research Centre, Parkwood Institute, St. Joseph’s Health Care London, London, Ontario Canada; 4 Department of Psychiatry, Western University, London, Ontario Canada

**Keywords:** Workplace mental health, Group randomized control trial, Intervention fidelity

## Abstract

**Background:**

Despite increased interest in workplace mental health interventions, the evidence for beneficial effects is mixed. Furthermore, many existing studies lack methodological rigor. We report results from a group randomized control trial to test the efficacy of a vastly popular intervention in Canada, the Road to Mental Readiness (R2MR) program, which has been widely disseminated in military, first responder, and civilian settings.

**Methods:**

The trial took place among Canadian Armed Forces military recruits completing their basic military qualification (BMQ) training, and randomized 65 platoons (*N* = 2831) into either (a) an Intervention (R2MR at week 2 of BMQ), or (b) a delayed Intervention Control (R2MR at week 9 of BMQ) condition. The principal investigator, participants, and data collection staff were blinded to platoon condition. Individual-level psychological functioning, resilience, mental health service use attitudes, intentions, and behaviours, and additional covariates were assessed with questionnaires around week 2 (a day or two before Intervention platoons received R2MR), at week 5, and at week 9 (a day or two before the Control platoons received R2MR). Military performance outcomes were obtained from administrative databases.

**Results:**

The full trial results were mixed; for some outcomes (psychological functioning, resilience, and military performance), we saw no evidence of beneficial effects; where we did see benefits (mental health service use attitudes, intentions, behaviours), the effects were very small, or disappeared over time. Analyses among two subsamples (Group 1: Intervention platoons with a Fidelity Check and their Controls, and Group 2: Intervention platoons without Fidelity Check and their Controls) indicated that for some outcomes (attitudes and help-seeking), under high fidelity conditions, the beneficial effects of R2MR were increased and better sustained; Conversely, under poor fidelity conditions, decreased beneficial effects or even iatrogenic effects were observed. Analyses across three training divisions indicated the larger organizational climate further influences efficacy.

**Conclusions:**

Our findings paint a very complex picture in which it is made evident that sensible, evidence-informed workplace mental health interventions such as R2MR may work under high fidelity conditions, but may yield no discernable benefit or even inadvertent iatrogenic effects if implemented poorly or without sufficient consideration to the larger organizational context.

**Trial registration:**

ISRCTN 52557050 Registered 13 October 2016.

## Background

Mental health problems constitute a global public health problem [1, 2] with significant economic costs [3]. The economic burden associated with mental health problems is driven in large part by costs associated with decreased productivity due to presenteeism and absenteeism. Over the past decade, there has been increasing interest in developing and implementing workplace mental health interventions to minimize these costs [4]. Nowhere have the interest and enthusiasm been greater than in military and public safety organizations where individuals are at an increased risk for developing mental health problems [5, 6] due to the stressful and oftentimes traumatogenic nature of their occupations.

Nevertheless, across civilian and non-civilian settings, the evidence for beneficial effects has been frustratingly mixed [4], with many studies failing to find consistent or sizeable beneficial effects; further, many existing studies in military settings suffer from significant methodological limitations [7], leading some to call into question the overall usefulness of such programs. The need for methodologically rigorous empirical tests of large workplace mental health interventions, especially in large military organizations that have been early and enthusiastic adopters of such programs, has never been greater.

Here we report findings from a group randomized control trial (GRCT) of a large workplace mental health intervention, the Road to Mental Readiness (R2MR) program, among military personnel in Canada. Developed by the Canadian Armed Forces (CAF) in 2007 [8], R2MR has since then been implemented across the deployment cycle and during career progression and has been delivered to a large number of military personnel. A 2015 report [9] estimated over 10,000 participants taking some version of R2MR annually in the CAF; those numbers are likely to be higher today. Furthermore, R2MR has been adapted by the Mental Health Commission of Canada (MHCC) for non-military, civilian and first responder work settings and has been delivered to an additional 100,000 participants and counting [10].

Surprisingly to date, there have been few empirical tests of the vastly popular R2MR program, which now has more than 30 distinct courses delivered during basic training and leadership courses, pre- and post- deployment training, occupation-specific training for certain occupations that may be at unique risk, performance coaching for instructors and supervisors, as well as training modules for military families. These courses have considerable overlap in content and delivery method but vary from 3 h to 5 days in duration, depending on the target audience. In CAF, data collected as part of routine program evaluation, immediately before and immediately after exposure to R2MR, seem to indicate increase in mental health literacy and decrease in stigma, as measured by items from the R2MR Program Evaluation Form (e.g., “If I have a mental health problem, there are things I can do to get better”, “I would be seen as weak if I sought help”), with effect sizes as high as 1.0 and .6, respectively [9], at least for the Basic Military Qualification (BMQ)/Basic Military Officer Qualification (BMOQ) versions of R2MR. However, whether such immediate improvements are sustained over time and whether they would be corroborated in methodologically rigorous, controlled studies is unknown. The MHCC reports beneficial effects for R2MR in decreasing stigma, and increasing self-reported resilience, both in first responder and civilian workplace settings, with effect sizes ranging from .12 to .65 [10]; unfortunately, these are once again based solely on data from routine program evaluation efforts and/or observational studies with pre-post designs, without comparison or control groups, or randomization. Interestingly, a recent observational study of R2MR among municipal police [11] found small (d = .29) beneficial effects in decreasing stigma at immediate post-training but these became non-significant at both 6- and 12-month follow-ups; further, there were no significant changes in mental health symptoms, resilience, or work engagement. Given its large-scale dissemination, and the well-known methodological limitations of the observational designs used in the few existing studies to test it, a more methodologically rigorous study of R2MR is overdue.

## Method

### Setting, participants, and sample size

This trial took place January 2017–May 2018 at the Canadian Forces Recruit and Leadership School (CFLRS), among Anglophone Non-Commissioned Member (NCM) recruits undergoing their 13-week BMQ training. R2MR at BMQ represents military personnel’s first exposure to the program. Recruits complete BMQ training within platoons of about 40–60 individuals; further, R2MR is delivered at the platoon level (i.e., there is a pre-existing grouping and clustering of intervention targets). Thus a group, rather than an individual randomized control trial was designed. A power analysis [12] estimated that 50–60 platoons were required to obtain sufficient statistical power for the full trial^1^.

### Trial design

The trial design was a GRCT in which platoons were randomly assigned to either an Intervention (R2MR at week 2 of BMQ) condition or a Delayed Intervention Control (R2MR at week 9 of BMQ) condition. At the time the trial was designed, all recruit platoons were receiving R2MR at week 2. Because R2MR had already become part of standard BMQ training, it was not possible to have a traditional, “pure”, control group that received no R2MR. Instead, a “Delayed Intervention” group that received R2MR close to the end of the BMQ, at week 9, served as the Control group.

The blocked randomization and allocation scheme was created by the civilian contractor/study biostatistician (A.L.) using Random Allocation Software - version 1.0 [15], with block sizes varying between 2 to 6. Compared to simple randomization, which does not guarantee equal numbers between study arms, blocked randomization has the advantage of ensuring that the number of platoons in intervention and control conditions are balanced at any stage of the trial [16, 17]. The randomization scheme^2^ was provided to the Scheduling Division of CFLRS so that the R2MR session, and the information and assessment sessions could be scheduled according to study design and the randomization scheme. The randomization scheme was also provided to the trial coordinator (K.B.) who checked and ensured on a weekly basis that the scheduled R2MR session and assessment sessions respected the study design and randomization scheme. The trial coordinator liaised between the biostatistician and the CFLRS scheduling division when there was a scheduling conflict, a missed session, or need to reschedule.

The trial was triple-blinded: The principal investigator (PI; D.F.), the participants, and the civilian contractors in charge of data collection did not have access to the randomization scheme and were blinded to platoon condition.

### Intervention details^3^

R2MR at BMQ has three objectives: 1) to increase mental health literacy, 2) to teach stress management skills, and 3) to change attitudes and intentions towards mental health service use (MHSU). R2MR uses a color-coded (green, yellow, orange, red) figure, the Mental Health Continuum Model (MHCM), to increase mental health literacy; a bidirectional arrow in the MHCM captures movement along the continuum, indicating that there is always the possibility for a return to full health and functioning; behavioral indicators under each color category in the MHCM familiarize recruits with basic mental health and mental illness concepts. To teach stress management skills, R2MR introduces four skills (i.e., the Big 4) to participants: tactical (diaphragmatic) breathing, goal-setting, visualization, and self-talk. Self-talk includes both positive mantras (repeating positive thoughts such as “I can do this”) and cognitive restructuring. After each skill is defined, the relevance of the Big 4 skills to successful military performance is addressed and recruits are given military-specific exercises to help practice the skills. Following the Big 4 skills, recruits learn how to recognize need for treatment using the MHCM; they are given information about what happens in treatment, and are provided with a list of resources available to individuals who might fall under each of the color categories in the MHCM. They are also presented with common attitudinal barriers to seeking treatment and provided with ways to overcome these barriers. After these didactic modules, recruits are broken into smaller groups, and are given hypothetical vignettes to help further reinforce mental health literacy and stress management skills. At BMQ, R2MR in its entirety is delivered to one platoon at a time, using standardized Powerpoint presentation slides, during a 160-min classroom session, by peer educators (typically former military members). All R2MR sessions in this GRCT were delivered by a single R2MR instructor. The R2MR sessions for the Intervention and the Control conditions used the same slides; the main difference was a slight adjustment in the speaker notes. The speaker notes for the Intervention condition which received R2MR at the beginning of the BMQ encouraged the use of the skills over the course of the BMQ, whereas the speaker notes for the Control condition which received R2MR towards the end of the BMQ emphasized the use of the skills over the course of one’s military career.

### Intervention Fidelity

Intervention fidelity can be quite complex [19], relating to not just well-adhered-to and competent delivery of the program by intervention staff, but also the program’s receipt (the extent to which key intervention concepts and skills are understood) and enactment (the extent to which key concepts and skills are used) by the target audience; further, even before a trial begins, fidelity with respect to study design and training of study staff must be carefully considered [20].

With respect to study design, a key concern was to prevent contamination between the Intervention and Control platoons. In discussions with CFLRS, we established that interaction between platoons during BMQ training is minimal. We also asked the recruit school to limit knowledge of the study and its design to a handful of staff in charge of scheduling to minimize the risk of unblinding and contamination. To ensure consistent dosing across all Intervention platoons, we used a standardized intervention manual developed by R2MR program staff, comprised of a set of Powerpoint slides, including suggested speaker points, with bolded points considered to be critical.

The trial instructor was selected by R2MR program administrators. The research team asked for an instructor with good overall instructional skills (e.g., establishing rapport) and open to additional training prior to the start of the trial. The instructor completed both standard training with R2MR program staff, plus additional training provided by the PI. The additional training of approximately 20 h spanned 4 months prior to the pilot phase of the trial and involved the PI i) observing several 160-min teaching sessions, ii) taking notes to help improve both adherence and competence, iii) completing a Fidelity Checklist, developed in prior research [21], and iv) meeting with the instructor immediately after the session to go over the notes taken on the manual as well as the completed Fidelity Checklist.

An independent observer (another peer educator/former military member) who would observe a small portion (approximately a fifth) of the trial’s intervention sessions was also selected by R2MR administrators and trained over several sessions by the PI to complete the Fidelity Checklist.

Interim (mid-trial) analyses [22] showed results were in the hypothesized direction only for the platoons that had received a Fidelity Check, and that for some outcomes the efficacy may have decreased over time, raising the possibility that i) when there was no observer in the room conducting a Fidelity Check, adherence and/or competence may have suffered, and that ii) the Fidelity Checklists as completed by the independent observer were not capturing increasing deviations by the instructor over time. These concerns were confirmed after the PI observed two Control platoon sessions-several deviations from standard speaker notes were noted, including omissions and insertion of new, contradictory material; the instructor was immediately retrained to standard. The PI also trained a new observer (A.N.) with a stronger background in R2MR concepts and skills (i.e., Ph.D. in Neuroscience) to complete the Fidelity Checklists for the remainder of the trial; as well, a decision was made to observe and complete Fidelity Checklists for all remaining Intervention platoons in the trial.

### Procedures^4^

Written Informed Consent was sought at an information session around week 2 of the BMQ, immediately followed by a baseline assessment (T1; for the Intervention platoons, a day or two before exposure to R2MR). During the information session, potential participants were told that all recruits would receive R2MR during the BMQ and that the study for which their consent was sought would examine the efficacy of R2MR by examining psychological health, resilience, attitudes, and performance at three points during the BMQ; there was no reference to the different (Intervention and Control) conditions in the trial. Follow-up assessments were conducted at approximately week 5 (T2) and week 9 (T3; For the Control platoons, a day or two before they received R2MR), of the BMQ respectively. Data were collected by civilian contractors, with Master’s or Ph.D.s in psychology or related fields, using standard scripts provided by the PI.

### Measures^5^

Key study outcomes, intermediate learning outcomes, and covariates were collected at the individual level.

#### Primary study outcomes

The main study outcomes were psychological functioning, resilience, and mental health service use (MHSU) attitudes, intentions, and behaviours. These were assessed at all three time-points (T1-T3) by the following tools: the Kessler Psychological Distress Scale (K-10) [25], the Subjective Units of Distress Scale (SUDS) [26], the Patient Health Questionnaire (PHQ) [27], Generalized Anxiety Disorder Scale (GAD-7) [28], the abbreviated Connor-Davidson Resilience Scale [29], and the CAF Mental Health Service Use Questionnaire (CAF-MHSUQ) [30]. The K-10 is a 10-item questionnaire assessing distress (nervousness, agitation, fatigue, and negative affect). Good internal consistency (α = .89 to .92) and construct validity have been established in the civilian and military samples [25, 31, 32]. The Subjective Units of Discomfort Scale (SUDS) [26] is a one-item self-report measure that assesses current subjective distress, anxiety, fear or discomfort on a scale from 0 to 100. Previous studies have shown preliminary evidence of satisfactory concurrent validity [26, 33]. The PHQ-9 is a brief, single factor, 9-item self-report questionnaire with well-established reliability, validity, and sensitivity [34–36]. The GAD-7 is a 1-factor, 7-item, self-report questionnaire with good internal consistency (α = .89) and validity in both the general population and primary care samples [28, 37, 38]. The CD-RISC has been widely used in community, clinical, and military samples to assess resilience and has demonstrated good internal consistency and construct validity for the original version [39], as well as the 10-item abbreviated version [29] we used. The Canadian Armed Forces Mental Health Service Use Questionnaire (CAF-MHSUQ) [30] is a 90-item self-report measure based on the Theory of Planned Behaviour [40] developed specifically to assess MHSU attitudes/ intentions among CAF recruits. Actual MHSU/help-seeking behaviours were assessed with an item asking whether the respondent had “seen or talked to i) friends, ii) family, iii) chaplain, iv) mental health nurse, v) social worker, vi) base surgeon, or vii) other person about problems with emotions, or mental health” during the BMQ and another item asking whether this was for the purposes of voluntary release.

The internal consistency reliability coefficients (Cronbach’s alpha) for the K-10, PHQ-9, GAD-7, and the CD-RISC were 0.89, 0.83, 0.87, and 0.85, respectively for T1, 0.92, 0.85, 0.89, and 0.91, respectively for T2, and 0.94, 0.89, 0.92, and 0.93, respectively for T3; for the CAF-MHSUQ Overall score and subscores of Instrumental Attitude, Affective Attitude, Subjective Norms, Perceived Self-Efficacy, Perceived Control, and Intentions, they were 0.89, 0.81, 0.90, 0.85, 0.78, and 0.90 respectively for T1, 0.91, 0.83, 0.92, 0.86, 0.79, and 0.91 respectively for T2, and 0.94, 0.87, 0.94, 0.88, 0.84, and 0.94, respectively for T3.

#### Secondary study outcomes

Secondary study outcomes were various indices of military performance. We used Graduation status as our primary military performance outcome. Information on Voluntary Release (VR) and intermediate military performance measures (e.g., results of the Fitness for Operational Requirements of CAF Employment (i.e., FORCE) test at week 1 and 8, the First Aid test, the Weapons Shooting test (out of a possible 25 points maximum, with minimum 15 point required for passing), were also obtained from a CFLRS administrative database after participants completed T3 assessments.

#### Intermediate R2MR learning outcomes

Intermediate R2MR learning outcomes of greater mental health literacy (MHL) and greater use of stress management skills are hypothesized to drive the presumed beneficial effects of R2MR. These were measured at T3 only. We measured MHL with items from the R2MR Program Evaluation Form [9], developed by the R2MR stakeholders to assess two aspects of mental health literacy, knowledge of basic mental health concepts and confidence in using available resources to help self and others when mental health issues do arise. We used the Test of Performance Strategies (TOPS) [41] to measure the frequency with which various stress managements skills taught in R2MR were used. The Cronbach’s alpha for the MHL and the TOPS subscores of Positive/Negative Thinking, Imagery, Goal setting, and Relaxation were 0.83, 0.77, 0.84, 0.87, and 0.85 respectively.

### Additional variables

#### Covariates

The following covariates were included only at Baseline/T1: A Sociodemographic Questionnaire developed specifically for this study to assess age, gender, ethnicity, education, and self-reported physical and mental health, the Shipley-2 [42] to assess cognitive aptitude, and the 33-item Marlowe-Crowne Social Desirability Scale (MC-SDS) [43] to assess socially desirable responding. The Cronbach’s alpha for the MC-SD at T1 was 0.64.

#### Cross-over

Similar to prior GRCTs on resilience training in military recruits [44], we used a single-item at T3 to ask participants the degree to which they talked with recruits in other platoons about R2MR. Responses were coded on a 4-point scale (1 = not at all to 5 = a great extent).

#### Fidelity checklist

Fidelity was measured using a checklist, developed in the context of a 4-year program of research on R2MR at BMQ [21], and assessing adherence to key intervention components, minor/major omissions, insertion of new/contradictory material, and time spent on the session.

### Hypotheses

Based on the existing literature [44–51] and previous research on R2MR among recruits [21, 52–59] (Fikretoglu, D., et al: Mental health education learning outcomes among Canadian Armed Forces recruits: examining the effects of intelligence, instructor type, and instruction type. Unpublished), we hypothesized that R2MR would have a beneficial effect on individual-level i) psychological functioning, ii) resilience, iii) MHSU attitudes, intentions, and behaviours, and iv) military performance. We further hypothesized that these beneficial effects would be driven by improvements in the intermediate learning outcomes of increased MHL and increased use of stress management skills. Based on prior research [4, 44, 45], we expected effect sizes in the very small-to-medium range, diminishing over time, from soon after post-intervention (T2) to short-term follow-up (T3).

### Statistical analyses

Some recruits pause their BMQ training due to not meeting fitness requirements, getting injured, or falling sick. Once ready to resume their training, these recruits may then be moved into a new platoon that is different than the platoon that they started their BMQ training in (i.e., they get “recoursed”). This poses obvious problems for GRCTs as recoursed recruits may move from Intervention to Control group (and vice versa). For this reason, we decided to remove recoursed recruits from the efficacy analyses, although we collected data from them. Additionally, a small number of platoons (approximately 10) start their BMQ training late in the calendar year and pause their training for 4 weeks during the Christmas Break to go home; they resume their BMQ training in early January. For some of these platoons, the break occurs between our trial’s T1 and T2 data collection sessions, for others, the break occurs between T2 and T3 sessions. Because we suspected that the efficacy of R2MR may be influenced by the larger context, we wanted to exclude these platoons from the efficacy analyses. However, we did this in a way that maximized sample size in each analysis (i.e., drop a platoon from the analysis if the outcome in question was assessed after Christmas Break; keep the platoon’s data for outcomes collected before the Christmas Break). In analyses on performance outcomes which were obtained from a CFLRS administrative database after all three self-report assessments were complete, platoons whose T3 data collection was conducted after the Christmas Break were dropped from analyses.

Data collected in this GRCT are clustered by platoon. We therefore used mixed linear models (assuming random intercepts and slopes to account for platoon-level variation) for continuous outcomes and generalized linear mixed models for binary outcomes to determine whether R2MR has beneficial effects on psychological health, resilience, MHSU attitudes/ intentions/behaviours, and military performance. The following individual-level variables were adjusted for in the models: baseline outcome, age, gender, ethnicity, education, self-reported physical health status, self-reported mental health status, K-10 score, SUDS score, GAD-7 score, PHQ-9 score, CD-RISC resilience score, the Shipley score, and the MC-SD social desirability score. The group/platoon-level variables of recourse rate, mean Shipley score, and mean MC-SD score were also adjusted for. All mixed linear models used inverse-probability-of-attrition-weighting to account for potential bias due to differential attrition.

We first ran the main efficacy analyses including all participants/platoons. Given the interim results [22], we then created two groups within the sample. Those Intervention platoons that had undergone an Intervention Fidelity Check by an independent observer and their control platoons from the same randomization block constituted Group 1 (With Fidelity Check). Group 2 (Without Fidelity Check), was comprised of the remaining Intervention platoons that had not undergone an Intervention Fidelity check and their Control platoons from the same randomization block. Importantly, given that the randomization and allocation were done by blocks, each of the two groups could be treated as an independent - albeit underpowered - GRCT. We re-assessed efficacy across the two groups using mixed linear models for continuous outcomes and generalized linear mixed models for binary outcomes.

Additional analyses looking at the effects of training division, as well as sensitivity analyses looking at whether including the recoursed recruits and the Christmas platoons would change the essence of the results were also conducted. Missingness rates were generally below 10%; participants with missing data were removed from analyses.

## Results

**Fig. 1:**
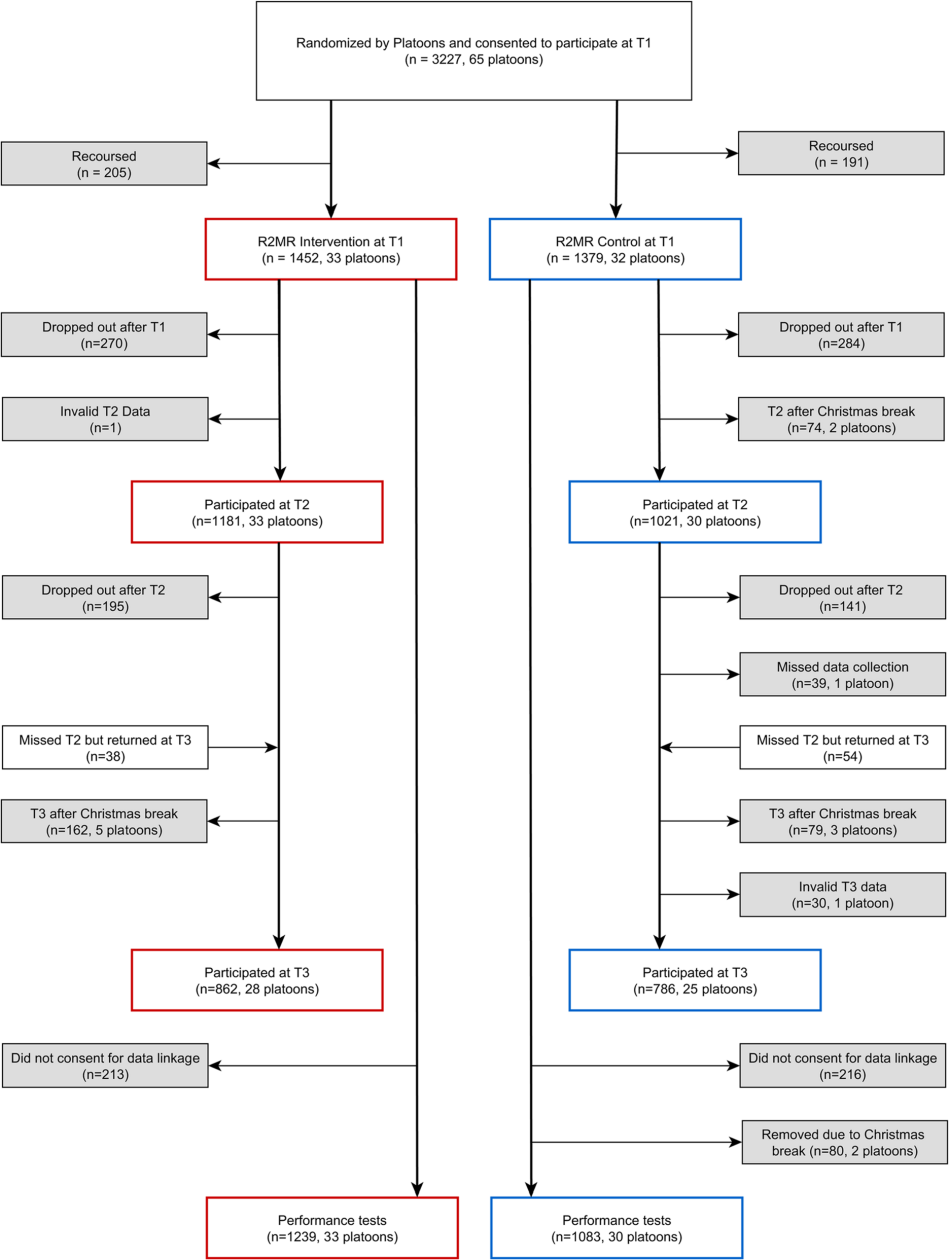
Participant and platoon flow through randomization to study condition and follow- up surveys, as well as final sample for analysis

Figure 1 captures the participant flow. Initially 67 platoons were included in the randomization scheme, of these one was cancelled and another did not show up for their T1 session. This left 65 platoons, with 33 randomized to the Intervention and 32 randomized to the Control condition. Two platoons that had their T2 and T3 data collection after Christmas Break were removed from all efficacy analyses. Eight platoons that had their T3 data collection after Christmas Break were removed from T3 efficacy analyses. One additional Control platoon was also removed from T3 efficacy analyses because its T3 data collection was mistakenly conducted after (not before) receiving R2MR. Thus, there were 65 platoons with a total of 2831 participants at Baseline/T1, 63 platoons with a total of 2202 participants at T2, and 53 platoons with a total of 1648 participants at T3 for self-report outcomes. For military performance outcomes obtained through linkage to an administrative database, 63 platoons with a total of 2322 participants provided data. Of the 33 Intervention platoons, 16 had undergone a Fidelity Check (8 by the first and another 8 by the second trained observer). Together with their 16 control platoons from the same randomization block, these platoons constituted Group 1 (With Fidelity Check). Group 2 (Without Fidelity Check), was comprised of the remaining 17 Intervention platoons and their 14 Control platoons.

### Descriptive statistics

Baseline participant characteristics are reported in Table 1. Overall, participants in both conditions reported mild-to-moderate distress, mild anxiety and depression, and moderate levels of resilience. They also reported slightly favorable attitudes and intentions towards MHSU. Intraclass correlation coefficients (ICCs), reported in Supplemental Table S5, were all very small (< 0.05), indicating little clustering.

**Table 1:** Baseline characteristics of study population by group allocation

	Intervention (*n* = 1452)	Control (*n* = 1379)
*Sex* (n,%)		
Male	1246, 86.53%	1154, 84.48%
Female	194, 13.47%	212, 15.52%
*Age* (mean, SD)	23.47, 5.51	23.38, 5.13
*Completed education* (n,%)		
Less than high school diploma or its equivalent	60, 4.16%	50, 3.67%
High school diploma or a high school equivalency certificate	833, 57.77%	755, 55.39%
Trade Certificate or Diploma	178, 12.34%	182, 13.35%
College, CEGEP, or other non-university certificate or diploma (other than trades certificate or diplomas)	254, 17.61%	260, 19.08%
University certificate or diploma below the bachelor’s level	21, 1.46%	18, 1.32%
Bachelor’s degree and University certificate or diploma above the bachelor’s level	96, 6.66%	98, 7.19%
*Ethnicity* (n,%)		
White	1200, 82.64%	1141, 82.74%
Other	252, 17.36%	238, 17.26%
*Self-reported general physical health* (n,%)		
Excellent	108, 7.49%	82, 6.02%
Very Good	423, 29.33%	382, 28.05%
Good	682, 47.30%	627, 46.04%
Fair	192, 13.31%	241, 17.69%
Poor	37, 2.57%	30, 2.20%
*Self-reported general mental health* (n,%)		
Excellent	230, 15.98%	178, 13.08%
Very Good	571, 39.68%	542, 39.82%
Good	484, 33.63%	483, 35.49%
Fair	129, 8.96%	136, 9.99%
Poor	25, 1.74%	22, 1.62%
*Shipley score* (mean, SD)	16.22, 4.61	16.08, 4.90
*Social desirability score* (mean, SD)	0.62, 0.21	0.61, 0.22
*K-10 total score* (mean, SD)	19.97, 7.07	20.41, 7.25
*SUDS score* (mean, SD)	43.02, 24.49	45.32, 24.53
*GAD total score* (mean, SD)	8.00, 5.34	8.43, 5.46
*PHQ-9 total score* (mean, SD)	6.55, 5.11	7.02, 5.18
*Resilience (CD-RISC) total score* (mean, SD)	28.91, 5.41	28.65, 5.33

### Lost to follow-up

A total of 2831 recruits consented to participate in the study and completed T1 data collection. Of those, 2276 completed T2 (80.40%) and 1963 completed T3 data collection (69.34%). Two attrition rates were calculated (T1-T2, and T1-T3). The overall attrition rate for T1–T2 was 19.60% and the overall attrition rate for T1–T3 was 30.66%.

### Cross-over

There was little communication about R2MR across platoons. Three quarters (75.73%) of the Control recruits reported “not at all” or “very little” communication, 20.49% reported discussing R2MR with other platoons “somewhat”, and very few (3.78%) reported discussing it “to a great extent”. The percentages for the Intervention recruits were similar (64.26, 26.64, and 9.10% for “not at all” or “very little”, “somewhat”, and “to a great extent”, respectively).

### Main efficacy analyses

At T2 (Table 2), there were no statistically significant differences between the Intervention and the Control groups on any of the psychological functioning or resilience scales. There were two trends for beneficial effects for MHSU Affective Attitudes and Self-Efficacy (d = 0.07 and 0.09, respectively). At T3 (Table 3), there were no statistically significant differences on any of the psychological functioning or resilience scales; the direction of the estimates was contrary to the hypothesized beneficial effects. There were no statistically significant differences on MHSU variables; in fact, most estimates were close to zero. There was a statistically significant beneficial effect for increased help-seeking from family and a trend for beneficial effects for increased help-seeking from chaplain/nurse/social worker/base surgeon. At T3, we also observed two trends for beneficial effects for intermediate learning outcomes of increased MHL and increased use of one stress management skill - relaxation (d = 0.06, and d = 0.10, respectively).

**Table 2:** Assessing R2MR efficacy at the 1st follow up

Outcomes	Difference between the intervention and control group		
	Estimates^a^	Cohen’s d	*p*-value
*Psychological functioning*			
K-10 total score	−0.01	–	0.98
SUDS score	0.31	–	0.78
GAD total score	−0.10	–	0.66
PHQ-9 total score	− 0.03	–	0.89
Resilience (CD-RISC) total score	−0.14	–	0.54
*Attitude (MHSU)*			
Instrumental attitude	0.09	–	0.12
Affective attitude	0.10	0.07	0.08
Intention	−0.01	–	0.82
Self-efficacy	0.10	0.09	0.07
Control	0.04	–	0.54
Subjective norms	0.01	–	0.79
Overall	0.06	–	0.16

^a^R2MR efficacy was assessed by the difference in the least squares means between the intervention and control group. The least squares means were calculated with the adjustment for baseline outcome, age, gender, ethnicity, education, self-reported physical health status, self-reported mental health status, K-10 score, SUDS score, GAD score, PhQ-9 score, resilience score, Shipley score, and social desirability score, platoon level mean Shipley score, platoon level mean social desirability score, and recourse rate. In addition, the calculation used inverse-probability-of-attrition-weights to account for the potential bias due to differential attrition

**Table 3:** Assessing R2MR efficacy at the 2nd follow up

Outcomes	Difference between the intervention and control group		
	Estimates	Cohen’s d	*p*-value
*Continuous outcomes* ^a^			
*Psychological functioning*			
K-10 total score	0.28	–	0.55
SUDS score	1.05	–	0.54
GAD total score	0.07	–	0.82
PHQ-9 total score	0.08	–	0.79
Resilience (CD-RISC) total score	−0.31	–	0.41
*Attitude (MHSU)*			
Instrumental attitude	0.06	–	0.37
Affective attitude	0.10	–	0.13
Intention	−0.08	–	0.26
Self-efficacy	0.09	–	0.18
Control	0.03	–	0.65
Subjective norms	0.05	–	0.42
Overall	0.04	–	0.46
*Mental Health Literacy*	0.06	0.09	0.07
*TOPS*			
Positive/negative thinking	0.03	–	0.56
Imagery	0.06	–	0.32
Goal setting	0.00	–	1.00
Relaxation	0.10	0.10	0.06
*Military Performance*			
Force test score at week 8	0.24	**–**	0.32
First aid test score	−0.01	**–**	0.98
Weapon test score	−0.50	**–**	0.19
*Binary outcomes* ^*b*^			
BMQ graduation^*c*^	0.71 (0.46–1.10)	**–**	0.13
Voluntary release^*d*^	1.16 (0.67–2.00)	**–**	0.60
*Help-seeking behavior* ^e^			
Chaplain/Nurse/SW/Surgeon	1.53 (0.95–2.46)	**–**	0.08
Other	0.70 (0.43–1.15)	**–**	0.16
None	0.86 (0.68–1.09)	**–**	0.22
Friends	1.18 (0.92–1.51)	**–**	0.20
Family	1.31 (1.03–1.66)	**–**	0.03

^a^ R2MR efficacy was assessed by the difference in the least squares means between the intervention and control group. The least squares means were calculated with the adjustment for baseline outcome, age, gender, ethnicity, education, self-reported physical health status, self-reported mental health status, K-10 score, SUDS score, GAD score, PhQ-9 score, resilience score, Shipley score, and social desirability score, platoon level mean Shipley score, platoon level mean social desirability score, and recourse rate. In addition, the calculation used inverse-probability-of-attrition-weights to account for the potential bias due to differential attrition

^b^ R2MR efficacy was assessed by the odds ratios contrasting the odds of success in the intervention group to the control group. The odds ratios (95%CI) were calculated from generalized linear mixed model with the adjustment for baseline outcome, age, gender, ethnicity, education, self-reported physical health status, self-reported mental health status, K-10 score, SUDS score, GAD score, PhQ-9 score, resilience score, Shipley score, and social desirability score**,** platoon level mean Shipley score, platoon level mean social desirability score, and recourse rate

^c^ BMQ graduation success rates were 89.07% in the intervention group and 90.96% in the control group

^d^ Voluntary release rates were 6.26% in the intervention group and 5.59% in the control group

^e^ Percentage of seeking help from Chaplain/Nurse/SW/Surgeon, Other, None, Friends, and Family were 6.68, 3.85, 38.38, 46.60, 54.04% in the intervention group and 3.89, 4.87, 40.19, 44.92, 49.24% in the control group

At T3 there were no statistically significant differences on the continuous military performance outcomes of Force Test score, First Aid Test score, and Weapons test score or the binary outcomes of BMQ graduation and VR.

Neither including the Christmas platoons nor including recoursed recruits in the analytic sample changed the essence of the main efficacy results (results available upon request from the first author).

### The effects of fidelity

These results, based on analyses which retained the effects of the original blocked randomization, are reported in Supplemental Tables S1-S2. For Group 1, for psychological functioning and resilience outcomes at T2, although non-significant, the findings were consistently in the direction of beneficial effects. In contrast, for Group 2, R2MR consistently showed negative effects contrary to hypothesized beneficial effects: Compared to the Control platoons, the Intervention platoons had higher psychological distress and lower resilience, although the results were not significant. At T3, there were few notable differences between Groups 1 and 2.

For Group 1, for MHSU outcomes at T2, although non-significant, all of the estimates were in the direction of beneficial effects for R2MR, with two (i.e., for Self-Efficacy, and Overall) reaching significance and one (for Affective Attitudes) showing a trend. In contrast, for Group 2, all but one estimate (for Instrumental Attitudes) were in the negative direction (i.e., the Intervention platoons reported more negative attitudes and intentions than the Control platoons) or near/at zero (i.e., no difference between Intervention and Control platoons). The differences between Groups 1 and 2 were even more striking at T3, with all MHSU estimates in the beneficial direction for Group 1 and almost all estimates in the negative direction for Group 2. Further we observed two statistically significant beneficial effects for Group 1 and in contrast, one statistically significant negative effect for Group 2. At T3, there were statistically significant beneficial effects for increased help-seeking from family and friends for Group 1; in contrast, these beneficial effects were absent for Group 2. The only exception to this pattern, for chaplain/nurse/social worker/base surgeon was most likely driven by extremely small sample sizes (24 for Group 1 and 20 for Group 2). For Group 1, almost all mental health literacy and stress management skills outcomes were in the direction of beneficial effects for R2MR at T3, with one outcome (MHL) reaching significance. For Group 2, although some estimates were in the direction of beneficial effects, others (including one for MHL) were close to zero, indicating an absence of beneficial effects.

For continuous BMQ military performance outcomes, there were no beneficial effects observed for either Group 1 or Group 2; Group 1 had one estimate and Group 2 had two estimates contrary to the hypothesized beneficial direction. For the binary outcomes of VR and BMQ graduation, for Group 1, the Odds Ratios (ORs) were around 1.00 indicating an absence of beneficial (or negative, harmful) effects. In contrast, for Group 2, the OR was .49 for BMQ graduation and statistically significant, indicating a negative, harmful effect (i.e., reduced likelihood of graduating from BMQ). For VR, the OR was close to 2.00 and trending toward significance, indicating increased likelihood of voluntarily releasing for the Intervention versus the Control group – again a negative, harmful effect for R2MR under conditions of no Fidelity Check.

### The effects of training division

Recruit platoons complete their BMQ in three training divisions within CFLRS. We wanted to examine in an exploratory fashion whether R2MR efficacy differed across different training divisions. These results are reported in Supplemental online Tables S3-S4. For MHSU attitudes, we observed estimates almost entirely in the hypothesized direction and at times reaching statistical significance in Division 1, and results partly or mostly contrary to hypothesized direction, and at times reaching statistical significance, in Divisions 2 and 3. For actual help-seeking behaviours, the pattern was different, with only Division 3 reporting statistically significant beneficial effects in the hypothesized direction. Sensitivity analyses, with samples limited to only Intervention platoons with Fidelity Check did not change the essence of the results.

## Discussion

We took a very popular workplace mental health intervention program and tested its efficacy. Overall, the full trial results were mixed; for some outcomes (psychological functioning, resilience, and military performance), we saw no evidence of beneficial effects; where we did see beneficial effects, the effects were very small and some (for MHSU variables) disappeared over time. This is consistent with prior research in both civilian and military settings [4, 7].

For psychological functioning, resilience, and military performance, there were no statistically significant beneficial effects, even under higher fidelity conditions. Beyond the obvious deleterious effects of poor fidelity on efficacy, we speculate the absence of beneficial effects for these outcomes may be partly explained by issues around receipt and enactment of key R2MR concepts and skills. Our prior research with military recruits found limited uptake of the Big 4 stress management skills, even under optimal fidelity conditions when the content was delivered by mental health professionals who developed R2MR [53]. In support of this, the current trial showed no statistically significant differences between the Intervention and the Control conditions in the use of the Big 4 skills at T3, with one exception for relaxation. The limited receipt and enactment findings are not surprising - basic military training environment is both physically and psychologically stressful and can undermine the learning and application of new skills; further, in the BMQ setting, R2MR is delivered in a single session. Distributed (multiple) sessions over time [44] and additional coaching of R2MR concepts and skills may yield better learning outcomes.

Our work extends the existing literature by showing that for some outcomes (MHSU attitudes and help-seeking), under high fidelity conditions, the efficacy of programs like R2MR may be increased - this is encouraging as it suggests these programs may yield beneficial effects for such outcomes when implemented with fidelity; conversely, however, our findings show that for some outcomes (MHSU attitudes, help-seeking, and military performance), under poor fidelity conditions, there may be decreased beneficial or even iatrogenic effects. Altogether, both high fidelity and low fidelity findings underscore the importance of adequately resourcing workplace mental health programs to carefully select, train, and continuously monitor program staff.

We are aware of no study to date looking at the effects of fidelity on efficacy in workplace mental health literature. Reviews of fidelity in psychotherapy research [60–62] have consistently found that few trials consider/assess it; when they do, they usually focus solely on delivery. The problem is even more pronounced in workplace mental health intervention research (Easterbrook, B., D. Fikretoglu, and Nazarov., A: Fidelity in workplace mental health intervention research: a narrative review and a prescriptive research agenda. Unpublished). Unfortunately, if fidelity is not assessed, we don’t know why a trial found null, negative, or mixed results, and may prematurely dismiss an otherwise promising intervention [20]. There are a number of obstacles to assessing fidelity [60–62]; we agree with others [60] that “CONSORT-style set of standards for [fidelity] reporting” (p. 230) would move workplace mental health research forward; routine reporting of fidelity and its effects on efficacy would also sensitize policy makers and program administrators to the importance of fidelity for their programs’ success.

Our trial also points to the importance of additional factors for program success. The finding of beneficial effects in one training division and no effect or somewhat iatrogenic effects in the other two training divisions underscores the importance of the larger organizational context for the success of workplace mental health interventions. Emerging implementation science research supports this view [63, 64]. Unfortunately, our trial did not have data that could inform which organizational/contextual factors may differ across training divisions and influence efficacy. Systematically examining organizational/contextual factors that may affect R2MR’s efficacy across different sets of key program outcomes is one focus of future R2MR research.

As pointed out by one of our reviewers, both the findings on the effects of fidelity and those on training division highlight the importance of conducting process evaluations, prior to or concurrently with randomized trials, using frameworks such as those advocated by The United Kingdom Medical Research Council [65] to look at three intersecting sets of factors that can influence the efficacy of an intervention: implementation factors such as fidelity and training of delivery staff, mechanism factors such as participant perceptions of and responses to the intervention, and contextual factors such as organizational climate that may be supportive or unsupportive of the intervention. Several factors, such as the fact that R2MR had already become part of standard BMQ training and the fact that the trial was taking place in a very busy military training establishment, precluded the inclusion of a process evaluation in this instance. However, we agree with others [65] that there needs to be increased awareness of the need for and the value of process evaluations in successfully implementing workplace mental health interventions such as R2MR.

## Conclusions

In interpreting our findings, we did not limit ourselves to looking at statistically significant differences. We noted the magnitude and the direction of the findings, highlighting results that were contrary to hypothesis, even when they were not statistically significant. This approach is in keeping with recommendations to note unexpected and/or negative results from RCTs [66] and limits the likelihood of certain types of reporting and citation biases in efficacy research, as recently noted in a review [67]. Our findings, when taken in their entirety, paint a very complex picture in which it is made evident that sensible, evidence-informed interventions that may work under certain conditions, may also produce inadvertent iatrogenic effects if implemented poorly or without sufficient consideration to the larger organizational, social-psychological context that may at the very least undermine efficacy. In the context of the widely-recognized limitations of RCTs [68], especially with respect to generalizability, the limitations of our own trial (e.g., the uniqueness of the basic military training context, the underpowered nature of the analyses looking at the effects of fidelity which were not pre-specified, the loss of the effects of randomization in analyses on training division), and the complexity of our findings, we refrain from strong recommendations for or against the R2MR program. Instead, we note the need for continued, rigorously-designed research - we are aware of at least two such efforts underway [10] - and increased sensitivity among researchers and program administrators alike to the importance of fidelity and organizational culture for the success of workplace mental health programs like R2MR. A limitation of our findings on the effects of fidelity was that we could only presume that when there was an observer in the classroom completing a fidelity check, there was better adherence to standard content than when there was no observer or fidelity check. While this is a reasonable assumption that seems to have been supported by data in our trial, as pointed out by one of our reviewers, it still remains that completion of a fidelity checklist does not automatically indicate high fidelity, nor does the absence of a checklist indicate poor fidelity. It will therefore be important for future trials of R2MR and similar workplace mental health interventions to more thoroughly measure and establish fidelity, using existing measuring tools as guides from recent literature [19, 69].

## Supplementary information

**Additional file 1: Table S1** Assessing R2MR efficacy at the 1st follow up among the fidelity (Group 1) and no fidelity check groups (Group 2). **Table S2** Assessing R2MR efficacy at the 2st follow up among the fidelity (Group 1) and no fidelity check groups (Group 2). **Table S3** Assessing R2MR efficacy at the 1st follow up among different divisions. **Table S4** Assessing R2MR efficacy at the 2st follow up among different divisions. **Table S5** Intraclass Correlation Coefficients (ICC, *k*) for T1 variables.

## Data Availability

The datasets generated and/or analysed during the current study are not publicly available in order to safeguard the privacy and confidentiality of information collected from military personnel but de-identified data may be available from the corresponding author on reasonable request once appropriate departmental approvals are obtained.

## Abbreviations

BMQ: Basic Military Qualification
CAF: Canadian Armed Forces
CAF-MHSUQ: Canadian Armed Forces Mental Health Service Use Questionnaire
CD-RISC: Connor-Davidson Resilience Scale
CFLRS: Canadian Forces Recruit and Leadership School
FORCE test: Fitness for Operational Requirements of CAF Employment Test
GAD-7: Generalized Anxiety Disorder Scale - 7
GRCT: Group Randomized Control Trial
ICC: Intraclass Correlation Coefficient
K-10: Kessler Psychological Distress Scale - 10
MC-SDS: Marlowe-Crowne Social Desirability Scale
MHCC: Mental Health Commission of Canada
MHCM: Mental Health Continuum Model
MHL: Mental Health Literacy
MHSU: Mental Health Service Use
NCM: Non-Commissioned Member
PHQ-9: Patient Health Questionnaire - 9
PI: Principal Investigator
R2MR: Road to Mental Readiness
SUDS: Subjective Units of Distress Scale
TOPS: Test of Performance Strategies
VR: Voluntary Release

## Footnotes

1 Power and sample size calculations were conducted with the Optimal Design Plus Empirical Evidence version 3.0 software [13] which is designed specifically for conducting power and sample size analyses in GRCTs. Required parameters including ICCs and average platoon (cluster) size were drawn initially from research leading up to the GRCT in our target population and updated [14] using data from the pilot phase of the GRCT. ICCs in the pilot GRCT ranged from 0 to 0.038. Average platoon size was estimated to be 28 taking into consideration a conservative participation rate of 50%. We assumed equal cluster size and 0 proportion of variance explained by group level covariates; Based on the existing literature, effect size was conservatively set at 0.2. The trial ended when estimated sample size was obtained. Further, this trial endpoint coincided with a significant change to BMQ training, a shortening of 2 weeks, which, had the trial continued to collect data, would have required a change in the third assessment point.

2 The randomization scheme took into consideration the fact that recruit platoons arrive at CFLRS in a staggered fashion over the course of the training year. For the 2016–17 and 2017–18 training years, the biostatistician was provided the training calendar several months ahead of time to generate the randomization scheme.

3 Intervention details have been taken from a previous publication on the R2MR program [18].

4 The trial procedures were piloted for feasibility with 10 platoons in late 2016, with close attention paid to randomization, scheduling, blinding, data collection (e.g., participation and retention rates), and data quality (e.g., missing data rates and patterns). Overall, the procedures were successfully implemented [23] minor modifications were made to data collection scripts to maximize participation and retention and to minimize missing data.

5 For measures with limited psychometric work or mixed results for construct/factorial validity (the CAF Mental Health Service Use Questionnaire (CAF-MHSUQ), the R2MR Program Evaluation Form for Mental Health Literacy (MHL), the Test of Performance Strategies (TOPS), and the Marlowe-Crown Social Desirability Scale (MC-SD)), we performed exploratory and confirmatory factor analyses with the pilot and the full trial data. Due to space limitations, these are captured in a separate report [24]. Scoring on these measures was based on the results from our psychometric analyses.

## References

[1] Whiteford HA (2013). Global burden of disease attributable to mental and substance use disorders: findings from the global burden of disease study 2010. Lancet.

[2] Whiteford HA (2015). The global burden of mental, neurological and substance use disorders: an analysis from the global burden of disease study 2010. PLoS One.

[3] Bloom DE (2011). The global economic burden of noncommunicable diseases.

[4] Vanhove AJ (2015). Can resilience be developed at work? A meta-analytic review of resilience-building programme effectiveness. J Occup Organ Psychol.

[5] Rusu C (2016). Prevalence comparison of past-year mental disorders and suicidal behaviours in the Canadian Armed Forces and the Canadian general population. Can J Psychiatr.

[6] Nasveld P (2012). Effects of deployment on mental health in modern military forces: a review of longitudinal studies. J Mil Vet Health.

[7] Vanhove AJ, Brutus T, Snowden KA (2018). Psychosocial health prevention programs in military organizations: a quantitative review of the evaluative rigor evidence. Occupational stress and well-being in military contexts.

[8] Zamorski MA (2016). The 2013 Canadian forces mental health survey: background and methods. Can J Psychiatr.

[9] Khan S. Road to Mental Readiness - Evaluation report. SGR-2014-009: Surgeon General Report/National Defence Health Services Group Ottawa, ON; 2015.

[10] Dobson KS (2018). Mental health initiatives in the workplace: models, methods, and results from the Mental Health Commission of Canada. World Psychiatry.

[11] Carleton RN (2018). A longitudinal assessment of the road to mental readiness training among municipal police. Cogn Behav Ther.

[12] Liu, A. and D. Fikretoglu, Power analysis for a proposed Group Randomized Control Trial (GRCT) on the Road to Mental Readiness (R2MR) program. DRDC-RDDC-2014-R068. Toronto, ON: Defence Research and Development Canada (DRDC; 2014.)

[13] Spybrook J (2011). Optimal design plus empirical evidence: documentation for the “optimal design” software version 3.0.

[14] Liu A, Fikretoglu D, Blackler K (2017). The estimated sample size needed for the group randomized control trial on the road to mental readiness: updated results from a second power analysis. DRDC-RDDC-2017-L177.

[15] Random Allocation Software version 1.0 April 15 2016]; Available from: http://mahmoodsaghaei.tripod.com/Softwares/randalloc.html.

[16] Efird J (2011). Blocked randomization with randomly selected block sizes. Int J Environ Res Public Health.

[17] Ivers NM, et al. Allocation techniques for balance at baseline in cluster randomized trials: a methodological review. Trials. 2012;13:120. 10.1186/1745-6215-13-120 PMC3503622 22853820

[18] Fikretoglu D (2017). Mental health and mental health service use attitudes among Canadian Armed Forces (CAF) recruits and officer cadets. DRDC-RDDC-2017-R027.

[19] Mars T (2013). Fidelity in complex behaviour change interventions: a standardised approach to evaluate intervention integrity. BMJ Open.

[20] Bellg AJ, et al. Enhancing treatment fidelity in health behavior change studies: best practices and recommendations from the NIH behavior change consortium. Health Psychol. ;2004(23):443–51. 10.1037/0278-6133.23.5.443 15367063

[21] Fikretoglu D, Lam Q, Beatty E (2013). Recommendations to improve treatment fidelity and the uptake of R2MR material prior to a group randomized control trial. DRDC Toronto LR-2013-10055-1-1404HD0500.

[22] Fikretoglu D (2018). The effects of intervention fidelity on efficacy in the group randomized control trial of the road to mental readiness program. DRDC-RDDC-2018-L165.

[23] Blackler K, Fikretoglu D, Liu A (2018). Feasibility findings from a pilot Group Randomized Control Trial on the Road to Mental Readiness (R2MR) program. DRCDC-RDDC-2018-R003.

[24] Frank C, Lee JEC, Fikretoglu D (2019). Validation of R2MR measurement tools among Canadian Armed Forces recruits. DRDC-RDDC-2019-L037.

[25] Kessler RC, et al. Short screening scales to monitor population prevalences and trends in non-specific psychological distress. Psychol Med. 2002;32(06):959–76. 10.1017/s0033291702006074 12214795

[26] Kaplan DM, Smith T, Coons J (1995). A validity study of the subjective unit of discomfort (SUD) score. Meas Eval Couns Dev.

[27] Spitzer RL (1999). Validation and utility of a self-report version of PRIME-MD: the PHQ primary care study. JAMA.

[28] Spitzer RL (2006). A brief measure for assessing generalized anxiety disorder: the GAD-7. Arch Intern Med.

[29] Campbell-Sills L, Stein MB (2007). Psychometric analysis and refinement of the connor–Davidson resilience scale (CD-RISC): validation of a 10-item measure of resilience. J Trauma Stress.

[30] Fikretoglu D, Blais A-R, Lam Q. Development and validation of a new theory of planned behavior questionnaire for mental health service use. DRDC-RDDC-2019-R111. Toronto, ON: Defence Research and Development Canada (DRDC; 2019).

[31] Blanc S (2014). How much distress is too much on deployed operations? Validation of the Kessler psychological distress scale (K10) for application in military operational settings. Mil Psychol.

[32] McFarlane A, Hodson SE (2011). Mental health in the Australian Defence Force: 2010 ADF mental health prevalence and wellbeing study: report. Department of Defence.

[33] Tanner BA (2012). Validity of global physical and emotional SUDS. Appl Psychophysiol Biofeedback.

[34] Kroenke K, Spitzer RL (2002). The PHQ-9: a new depression diagnostic and severity measure. Psychiatr Ann.

[35] Kroenke K, Spitzer RL, Williams JB (2001). The PHQ-9. J Gen Intern Med.

[36] Cameron IM (2008). Psychometric comparison of PHQ-9 and HADS for measuring depression severity in primary care. Br J Gen Pract.

[37] Löwe B (2008). Validation and standardization of the generalized anxiety disorder screener (GAD-7) in the general population. Med Care.

[38] Ruiz MA (2011). Validity of the GAD-7 scale as an outcome measure of disability in patients with generalized anxiety disorders in primary care. J Affective Disord.

[39] Connor KM, Davidson JR (2003). Development of a new resilience scale: the Connor-Davidson resilience scale (CD-RISC). Depress and Anxiety.

[40] Ajzen I (1991). The theory of planned behavior. Organ Behav Hum Decis Process.

[41] Thomas PR, Murphy SM, Hardy L (1999). Test of performance strategies: development and preliminary validation of a comprehensive measure of athletes' psychological skills. J Sports Sci.

[42] Shipley WC (2009). Shipley-2 manual.

[43] Crowne DP, Marlowe D (1960). A new scale of social desirability independent of psychopathology. J Consult Psychol.

[44] Adler AB (2015). Resilience training with soldiers during basic combat training: randomisation by platoon. Appl Psychol Health Well Being..

[45] Adler AB (2009). Battlemind debriefing and battlemind training as early interventions with soldiers returning from Iraq: randomization by platoon. J Consult Psychol.

[46] Adler AB, et al. Mental skills training with basic combat training soldiers: a group-randomized trial. J Appl Psychol. 2015;100(6):1752–64. 10.1037/apl0000021 26011718

[47] Castro C (2012). Mental health training with soldiers four months after returning from Iraq: randomization by platoon. J Trauma Stress.

[48] Cohn A, Pakenham K (2008). Efficacy of a cognitive-behavioral program to improve psychological adjustment among soldiers in recruit training. Mil Med.

[49] Mulligan K (2012). Postdeployment Battlemind training for the U.K. armed forces: a cluster randomized controlled trial. J Consult Clin Psychol.

[50] Williams A (2007). STARS: Stratgeies to assist navy recruits' success. Mil Med.

[51] Williams A (2004). Psychosocial effects of the boot strap intervention in navy recruits. Mil Med.

[52] Fikretoglu D, Beatty E, Liu A. Comparing different versions of R2MR to determine optimal content: testing instruction type, homework, and intelligence effects at two timepoints. DRDC Toronto R14–1017-1237. Toronto: DRDC; 2014.

[53] Fikretoglu D, Beatty E, Liu A (2014). Optimizing R2MR at basic military qualification (BMQ): lessons learned from studies conducted between 2012 and 2014 and recommendations for implementation. DRDC-RDDC-2014-L244.

[54] Muller-Gass, A., et al., Assessing daytime sleepiness and fatigue in recruits at the Canadian forces leadership and recruit school. DRDC-RDDC 2015 R118, D. Toronto, Editor. 2015, DRDC Toronto.

[55] Jobidon ME, et al. Attitudes des recrues Francophones de L’école de Leadership et de Recrues des Forces Canadiennes envers le recours aux services de santé mentale: Résultants et implications de la validation de la version française de questionnaire: Toronto-LR-2013-10055-1-1404HD0500 to Directorate General Health Services, Directorate of Mental Health.Toronto: DRBC; 2013. p. 2013.

[56] Lee, J. et al. Mental health services use intentions among Canadian military recruits. Mil Psychol 2016; 28(6), 498-505.

[57] Fikretoglu D, Lam Q, Blais A-R. Attitudes towards mental health service use among CAF recruits during basic military qualification: research findings and specific recommendations for improving the related R2MR content for recruits: DRDC Toronto-LR-2013-10055-1-1404HD0500 to directorate general health services, Directorate of Mental Health. Toronto: DRDC; 2013. p. 2013.

[58] Fikretoglu D, Beatty E, Liu A (2014). Comparing different versions of road to mental readiness to determine optimal content: testing instruction type, homework, and intelligence effects at two timepoints. DRDC-RRDC-2014-R164.

[59] Fikretoglu D, Liu A, Blackler K (2016). Testing different methods to optimize change in mental health service use attitudes: findings and recommendations for the road to mental readiness (R2MR) program at basic military qualification. DRDC-RDDC-2016-R025.

[60] Cox JR, Martinez RG, Southam-Gerow MA (2019). Treatment integrity in psychotherapy research and implications for the delivery of quality mental health services. J Consult Clin Psychol.

[61] Perepletchikova F, Kazdin AE (2005). Treatment integrity and therapeutic change: issues and research recommendations. Clinical Psychol.

[62] Perepletchikova F, Treat TA, Kazdin AE (2007). Treatment integrity in psychotherapy research: analysis of the studies and examination of the associated factors. J Consult Clin Psychol.

[63] Williams NJ, Beidas RS. Annual research review: the state of implementation science in child psychology and psychiatry: a review and suggestions to advance the field. J Child Psychol Psychiatry. 2018. 10.1111/jcpp.12960. 10.1111/jcpp.12960 PMC6389440 30144077

[64] Williams NJ, et al. Organizational culture and climate profiles: relationships with fidelity to three evidence-based practices for autism in elementary schools. Implement Sci. 2019. 10.1186/s13012-019-0863-9. 10.1186/s13012-019-0863-9 PMC6373074 30755220

[65] Moore GF (2015). Process evaluation of complex interventions: Medical Research Council guidance. BMJ.

[66] Hewitt CE, Mitchell N, Torgerson DJ (2008). Heed the data when results are not significant. BMJ.

[67] de Vries YA (2018). The cumulative effect of reporting and citation biases on the apparent efficacy of treatments: the case of depression. Psychol Med.

[68] Deaton A, Cartwright N (2018). Understanding and misunderstanding randomized control trials. Soc Sci Medi.

[69] Toomey E, Matthews J, Hurley DA (2017). Using mixed methods to assess fidelity of delivery and its influencing factors in a complex selfmanagement intervention for people with osteoarthritis and low back pain. BMJ Open.

