# Supplementary material for: A group randomized control trial to test the efficacy of the Road to Mental Readiness (R2MR) program among Canadian military recruits
Source: BMC Psychiatry. 2019 Oct 29;19:326. doi: 10.1186/s12888-019-2287-0 (PMC6819517; doi:10.1186/s12888-019-2287-0)
Supplement: Supplementary file 1 — Additional file 1: Table S1 Assessing R2MR efficacy at the 1st follow up among the fidelity (Group 1) and no fidelity check groups (Group 2). Table S2 Assessing R2MR efficacy at the 2st follow up among the fidelity (Group 1) and no fidelity check groups (Group 2). Table S3 Assessing R2MR efficacy at the 1st follow up among different divisions. Table S4 Assessing R2MR efficacy at the 2st follow up among different divisions. Table S5 Intraclass Correlation Coefficients (ICC, k) for T1 variables. [file 12888_2019_2287_MOESM1_ESM.docx]

Supplemental Online Table 1 (S1). Assessing R2MR efficacy at the 1^st^ follow up among the fidelity (Group 1) and no fidelity check groups (Group 2)

| J | Difference between the intervention and control group among the fidelity check group | | | | Difference between the intervention and control group among the no fidelity check group | | | |
| --- | --- | --- | --- | --- | --- | --- | --- | --- |
|  | Estimates^1^ | Cohen’s d | p-value | Estimates^1^ | | Cohen’s d | p-value |  |
| *Psychological functioning* |  |  |  |  | |  |  |  |
| K-10 total score | -0.39 | - | 0.54 | 0.29 | | - | 0.52 |  |
| SUDS score | -1.52 | - | 0.36 | 1.49 | | - | 0.31 |  |
| GAD total score | -0.18 | - | 0.63 | 0.06 | | - | 0.85 |  |
| PHQ-9 total score | -0.33 | - | 0.33 | 0.26 | | - | 0.45 |  |
| Resilience (CD-RISC) total score | 0.12 | - | 0.77 | -0.41 | | - | 0.15 |  |
| *Attitude (MHSU)* |  |  |  |  | |  |  |  |
| Instrumental attitude | 0.08 | - | 0.29 | 0.14 | | - | 0.17 |  |
| Affective attitude | 0.15 | 0.16 | 0.09 | 0.07 | | - | 0.41 |  |
| Intention | 0.04 | - | 0.52 | -0.05 | | - | 0.50 |  |
| Self-efficacy | 0.19 | 0.10 | 0.01 | 0.00 | | - | 0.98 |  |
| Control | 0.10 | - | 0.20 | 0.00 | | - | 0.99 |  |
| Subjective norms | 0.09 | - | 0.13 | -0.05 | | - | 0.52 |  |
| Overall | 0.11 | 0.12 | 0.04 | 0.02 | | - | 0.78 |  |

^1^ R2MR efficacy was assessed by the difference in the least squares means between the intervention and control group. The least squares means were calculated with the adjustment for baseline outcome, age, gender, ethnicity, education, self-reported physical health status, self-reported mental health status, K-10 score, SUDS score, GAD score, PhQ-9 score, resilience score, Shipley score, and social desirability score, platoon level mean Shipley score, platoon level mean social desirability score, and recourse rate. In addition, the calculation used inverse-probability-of-attrition-weights to account for the potential bias due to differential attrition.

Supplemental Online Table 2 (S2). Assessing R2MR efficacy at the 2^st^ follow up among the fidelity (Group 1) and no fidelity check groups (Group 2)

|  | Difference between the intervention and control group among the fidelity check group | | | | Difference between the intervention and control group among the no fidelity check group | | | | |
| --- | --- | --- | --- | --- | --- | --- | --- | --- | --- |
|  | Estimates | Cohen’s d | p-value | Estimates | | Cohen’s d | p-value |  |  |
| Continuous outcome^1^ | | | | | | | | |  |
| *Psychological functioning* |  |  |  |  | |  |  |  |  |
| K-10 total score | 0.08 | - | 0.91 | 0.47 | | - | 0.45 |  |  |
| SUDS score | 1.17 | - | 0.65 | 0.79 | | - | 0.74 |  |  |
| GAD total score | 0.06 | - | 0.88 | 0.09 | | - | 0.84 |  |  |
| PHQ-9 total score | 0.17 | - | 0.71 | -0.01 | | - | 0.98 |  |  |
| Resilience (CD-RISC) total score | 0.01 | - | 0.98 | -0.58 | | - | 0.26 |  |  |
| *Attitude (MHSU)* |  |  |  |  | |  |  |  |  |
| Instrumental attitude | 0.14 | - | 0.15 | 0.00 | | - | 0.98 |  |  |
| Affective attitude | 0.11 | - | 0.23 | 0.11 | | - | 0.26 |  |  |
| Intention | 0.04 | - | 0.59 | -0.24 | | -0.16 | 0.05 |  |  |
| Self-efficacy | 0.19 | 0.16 | 0.04 | -0.04 | | - | 0.68 |  |  |
| Control | 0.10 | - | 0.23 | -0.03 | | - | 0.75 |  |  |
| Subjective norms | 0.16 | 0.15 | 0.03 | -0.08 | | - | 0.35 |  |  |
| Overall | 0.10 | 0.11 | 0.10 | -0.04 | | - | 0.59 |  |  |
| *Mental Health Literacy* | 0.10 | 0.16 | 0.03 | 0.04 | | - | 0.40 |  |  |
| *TOPS* |  |  |  |  | |  |  |  |  |
| Positive/negative thinking | 0.08 | - | 0.19 | 0.00 | | - | 0.99 |  |  |
| Imagery | 0.05 | - | 0.53 | 0.09 | | - | 0.30 |  |  |
| Goal setting | -0.01 | - | 0.89 | 0.03 | | - | 0.71 |  |  |
| Relaxation | 0.11 | - | 0.14 | 0.14 | | 0.15 | 0.06 |  |  |
| *Military Performance* |  |  |  |  | |  |  |  |  |
| Force test score at week 8 | 0.34 | - | 0.41 | 0.21 | | - | 0.37 |  |  |
| First aid test score | 0.60 | - | 0.50 | -0.55 | | - | 0.17 |  |  |
| Weapon test score | -0.48 | - | 0.44 | -0.41 | | - | 0.39 |  |  |
| Binary outcomes^2^ | | | | | | | | |  |
| BMQ graduation*^3^* | 0.92 (0.51 - 1.64) | - | 0.77 | 0.49 (0.25 - 0.99) | | - | 0.05 |  |  |
| Voluntary release*^4^* | 0.90 (0.42 - 1.94) | - | 0.78 | 1.82 (0.82 - 4.05) | | - | 0.14 |  |  |
| *Help-seeking behavior^5^* |  |  |  |  | |  |  |  |  |
| Chaplain/Nurse/SW/Surgeon | 1.07 (0.52 - 2.17) | - | 0.86 | 2.77 (1.32 - 5.82) | | - | 0.01 |  |  |
| Other | 0.71 (0.33 - 1.51) | - | 0.37 | 0.74 (0.38 - 1.46) | | - | 0.39 |  |  |
| None | 0.69 (0.50 - 0.95) | - | 0.02 | 1.13 (0.79 - 1.63) | | - | 0.51 |  |  |
| Friends | 1.67 (1.22 - 2.29) | - | 0.002 | 0.82 (0.58 - 1.17) | | - | 0.27 |  |  |
| Family | 1.76 (1.28 - 2.41) | - | 0.0005 | 0.90 (0.63 - 1.28) | | - | 0.55 |  |  |

^1^ R2MR efficacy was assessed by the difference in the least squares means between the intervention and control group. The least squares means were calculated with the adjustment for baseline outcome, age, gender, ethnicity, education, self-reported physical health status, self-reported mental health status, K-10 score, SUDS score, GAD score, PhQ-9 score, resilience score, Shipley score, and social desirability score, platoon level mean Shipley score, platoon level mean social desirability score, and recourse rate. In addition, the calculation used inverse-probability-of-attrition-weights to account for the potential bias due to differential attrition.

^2^ R2MR efficacy was assessed by the odds ratios contrasting the odds of success in the intervention group to the control group. The odds ratios (95%CI) were calculated from generalized linear mixed model with the adjustment for baseline outcome, age, gender, ethnicity, education, self-reported physical health status, self-reported mental health status, K-10 score, SUDS score, GAD score, PhQ-9 score, resilience score, Shipley score, and social desirability score**,** platoon level mean Shipley score, platoon level mean social desirability score, and recourse rate.

^3^ BMQ graduation success rates were 88.98% and 88.67% in the intervention and control groups of the fidelity check group. The rates were 89.17% and 94.14% in the intervention and control groups of the no fidelity check group.

^4^ Voluntary release rates were 6.57% and 7.20% in the intervention and control groups of the fidelity check group. The rates were 5.94% and 3.35% in the intervention and control groups of the no fidelity check group.

^5^ In the fidelity check group, percentage of seeking help from Chaplain/Nurse/SW/Surgeon, Other, None, Friends, and Family were 5.65%, 3.29%, 36.71%, 48.00%, 55.29% in the intervention group and 4.96%, 3.97%, 43.42%, 39.70%, 44.17% in the control group. In the no fidelity check group, percentage of seeking help from Chaplain/Nurse/SW/Surgeon, Other, None, Friends, and Family were 7.91%, 4.52%, 40.40%, 44.92%, 52.54% in the intervention group and 2.53%, 6.01%, 36.08%, 51.58%, 55.70% in the control group.

Supplemental Online Table 3 (S3). Assessing R2MR efficacy at the 1^st^ follow up among different divisions

|  | Difference between the intervention and control group among Division 1 | | Difference between the intervention and control group among Division 2 | | Difference between the intervention and control group among Division 3 | |
| --- | --- | --- | --- | --- | --- | --- |
|  | Estimates^1^ | p-value | Estimates^1^ | p-value | Estimates^1^ | p-value |
| *Psychological functioning* |  |  |  |  |  |  |
| K-10 total score | -0.31 | 0.68 | 0.18 | 0.87 | 0.07 | 0.90 |
| SUDS score | -1.03 | 0.62 | 1.10 | 0.68 | 0.57 | 0.75 |
| GAD total score | -0.05 | 0.92 | 0.04 | 0.95 | -0.14 | 0.69 |
| PHQ-9 total score | 0.14 | 0.75 | 0.44 | 0.46 | -0.18 | 0.70 |
| Resilience (CD-RISC) total score | 0.04 | 0.94 | -0.50 | 0.24 | 0.20 | 0.62 |
| *Attitude (MHSU)* |  |  |  |  |  |  |
| Instrumental attitude | 0.20 | 0.14 | 0.01 | 0.92 | 0.11 | 0.26 |
| Affective attitude | 0.25 | 0.050 | 0.08 | 0.57 | 0.01 | 0.90 |
| Intention | 0.12 | 0.23 | -0.04 | 0.73 | -0.03 | 0.73 |
| Self-efficacy | 0.19 | 0.09 | 0.06 | 0.59 | 0.08 | 0.41 |
| Control | 0.08 | 0.48 | 0.09 | 0.43 | -0.10 | 0.33 |
| Subjective norms | 0.12 | 0.34 | -0.05 | 0.64 | -0.06 | 0.53 |
| Overall | 0.15 | 0.051 | 0.02 | 0.82 | -0.01 | 0.90 |

^1^ R2MR efficacy was assessed by the difference in the least squares means between the intervention and control group. The least squares means were calculated with the adjustment for baseline outcome, age, gender, ethnicity, education, self-reported physical health status, self-reported mental health status, K-10 score, SUDS score, GAD score, PhQ-9 score, resilience score, Shipley score, and social desirability score, platoon level mean Shipley score, platoon level mean social desirability score, and recourse rate. In addition, the calculation used inverse-probability-of-attrition-weights to account for the potential bias due to differential attrition.

Supplemental Online Table 4 (S4). Assessing R2MR efficacy at the 2^st^ follow up among different divisions

|  | Difference between the intervention and control group among Division 1 | | Difference between the intervention and control group among Division 2 | | Difference between the intervention and control group among Division 3 | |
| --- | --- | --- | --- | --- | --- | --- |
|  | Estimates | p-value | Estimates | p-value | Estimates | p-value |
| Continuous outcomes^1^ | | | | | | |
| *Psychological functioning* |  |  |  |  |  |  |
| K-10 total score | 0.82 | 0.20 | 0.78 | 0.56 | 0.97 | 0.32 |
| SUDS score | 0.72 | 0.76 | 4.70 | 0.30 | 2.91 | 0.42 |
| GAD total score | -0.09 | 0.84 | 0.35 | 0.61 | 0.70 | 0.27 |
| PHQ-9 total score | 0.53 | 0.16 | 0.66 | 0.43 | 0.40 | 0.47 |
| Resilience (CD-RISC) total score | 0.23 | 0.73 | -0.17 | 0.77 | -1.42 | 0.09 |
| *Attitude (MHSU)* |  |  |  |  |  |  |
| Instrumental attitude | 0.10 | 0.52 | -0.08 | 0.59 | 0.06 | 0.63 |
| Affective attitude | -0.004 | 0.98 | 0.12 | 0.37 | 0.03 | 0.80 |
| Intention | 0.14 | 0.46 | -0.27 | 0.04 | -0.20 | 0.14 |
| Self-efficacy | 0.26 | 0.09 | -0.27 | 0.03 | 0.05 | 0.61 |
| Control | 0.29 | 0.01 | -0.10 | 0.43 | -0.18 | 0.10 |
| Subjective norms | 0.23 | 0.12 | -0.08 | 0.53 | -0.05 | 0.64 |
| Overall | 0.19 | 0.13 | -0.17 | 0.06 | -0.06 | 0.50 |
| *Mental Health Literacy* | 0.18 | 0.01 | -0.05 | 0.47 | -0.04 | 0.52 |
| *TOPS* |  |  |  |  |  |  |
| Positive/negative thinking | -0.05 | 0.48 | 0.05 | 0.72 | -0.02 | 0.83 |
| Imagery | 0.07 | 0.61 | 0.03 | 0.79 | 0.00 | 1.00 |
| Goal setting | 0.08 | 0.52 | 0.05 | 0.63 | -0.07 | 0.44 |
| Relaxation | 0.18 | 0.16 | 0.08 | 0.47 | -0.07 | 0.43 |
| *Military Performance* |  |  |  |  |  |  |
| Force test score at week 8 | -0.05 | 0.91 | 0.60 | 0.15 | 0.59 | 0.21 |
| First aid test score | 1.88 | 0.02 | -0.89 | 0.14 | -0.80 | 0.42 |
| Weapon test score | 0.27 | 0.67 | -3.18 | 0.001 | -0.26 | 0.64 |
| Binary outcomes^2^ | | | | | | |
| BMQ graduation*^3^* | 1.12 (0.45 - 2.78) | 0.81 | 0.28 (0.11 - 0.72) | 0.01 | 0.95 (0.49 - 1.81) | 0.86 |
| Voluntary release*^4^* | 0.83 (0.28 - 2.46) | 0.73 | 2.28 (0.52 - 9.99) | 0.27 | 1.18 (0.44 - 3.15) | 0.74 |
| *Help-seeking behavior^5^* |  |  |  |  |  |  |
| None | 0.89 (0.56 - 1.41) | 0.61 | 1.02 (0.60 - 1.73) | 0.95 | 0.65 (0.42 - 1.00) | 0.05 |
| Friends | 1.07 (0.69 - 1.65) | 0.76 | 0.95 (0.54 - 1.67) | 0.85 | 1.58 (1.04 - 2.40) | 0.03 |
| Family | 1.29 (0.84 - 1.99) | 0.25 | 1.11 (0.65 - 1.89) | 0.71 | 1.97 (1.30 - 2.99) | 0.001 |

^1^ R2MR efficacy was assessed by the difference in the least squares means between the intervention and control group. The least squares means were calculated with the adjustment for baseline outcome, age, gender, ethnicity, education, self-reported physical health status, self-reported mental health status, K-10 score, SUDS score, GAD score, PhQ-9 score, resilience score, Shipley score, and social desirability score, platoon level mean Shipley score, platoon level mean social desirability score, and recourse rate. In addition, the calculation used inverse-probability-of-attrition-weights to account for the potential bias due to differential attrition.

^2^ R2MR efficacy was assessed by the odds ratios contrasting the odds of success in the intervention group to the control group. The odds ratios (95%CI) were calculated from generalized linear mixed model with the adjustment for baseline outcome, age, gender, ethnicity, education, self-reported physical health status, self-reported mental health status, K-10 score, SUDS score, GAD score, PhQ-9 score, resilience score, Shipley score, and social desirability score**,** platoon level mean Shipley score, platoon level mean social desirability score, and recourse rate.

^3^ BMQ graduation success rates were 87.37% and 86.36% in the intervention and control groups of Division 1. The rates were 86.36% and 93.91% in the intervention and control groups of Division 2. The rates were 92.50% and 90.63% in the intervention and control groups of Division 3.

^4^ Voluntary release rates were 7.24% and 6.79% in the intervention and control groups of Division 1. The rates were 6.90% and 5.01% in the intervention and control groups of Division 2. The rates were 4.70% and 5.52% in the intervention and control groups of Division 3.

^5^ Chaplain/Nurse/SW/Surgeon and Other categories were removed due to insufficient sample size for regression analysis. In Division 1, percentage of seeking help from None, Friends, and Family were 37.04%, 48.72%, 54.42% in the intervention group; and 39.08%, 47.70%, 48.85% in the control group. In Division 2, percentage of seeking help from None, Friends, and Family were 37.69%, 44.62%, 56.15% in the intervention group; and 37.54%, 47.32%, 53.94% in the control group. In Division 3, percentage of seeking help from None, Friends, and Family were 40.27%, 44.97%, 52.68% in the intervention group; and 44.74%, 39.47%, 42.98% in the control group.

Supplemental Online Table 5 (S5). Intraclass Correlation Coefficients (ICC, *k*) for T1 variables

| *Variables* | *ICC* |
| --- | --- |
| Shipley score | 0.017 |
| Social desirability total score | 0.007 |
| K10 total score | 0.009 |
| SUDS score | 0.026 |
| GAD total score | 0.028 |
| PHQ-9 total score | 0.021 |
| Resilience (CDRISC) total score | 0.007 |
| Attitude |  |
| Instrumental attitude | 0.007 |
| Affective attitude | 0.000 |
| Intention | 0.010 |
| Self-efficacy | 0.016 |
| Control | 0.015 |
| Subjective norms | 0.012 |
| Overall | 0.011 |
